# More injury stoppage time in women compared to men in elite level football tournaments: Retrospective comparison of match injuries in 2018 FIFA Men’s World Cup and 2019 FIFA Women’s World Cup

**DOI:** 10.5114/biolsport.2025.139854

**Published:** 2024-08-08

**Authors:** Gürhan Dönmez, Ömer Faruk İlicepınar, Şerife Şeyma Torgutalp, Karim Chamari

**Affiliations:** 1 Hacettepe University Department of Sports Medicine, Turkey; 2 Gençlerbirliḡi Sports Club, Ankara, Turkey; 3 Mardin Training and Research Hospital Sports Medicine Department, Mardin, Turkey; 4 Naufar, Wellness and Recovery Center, Doha, Qatar; 5 Higher institute of Sport and Physical Education, ISSEP Ksar Saïd, Manouba University, Tunis, Tunisia

**Keywords:** Injury, Incidence, World cup, Football, Team physician

## Abstract

To identify the incidence of injury time-out due to field injuries in highest level professional football (soccer) international tournaments and to compare the features of these incidents between men’s and women’s football. The incidence of stoppage time due to incidents and the resulting injury characteristics of professional football players participating in the 2018 FIFA Men’s World Cup (MWC) in Russia and the 2019 FIFA Women’s World Cup (WWC) in France were examined retrospectively through video analysis. In the 2018 MWC, a total of 123 injury time-outs with 132 treatments occurred in 64 matches, while in the 2019 WWC, 142 incidents with 150 injured players were recorded in 52 matches. The incidence of stoppage time was higher in women than in men (81.2 vs. 56.8 per 1000 match hours, respectively, p = 0.004), and accordingly, women had a higher overall incidence of injury (IRR = 1.4 [95%CI = 1.1–1.8], p = 0.005). Despite women required more medical care during games, the substitution rate after the incidents was higher for men (27.3% to 15.3%, p = 0.02). Almost three-quarters of incidents for both sexes were sudden-onset contact injuries and the most common site was the lower limb. Although more frequent match incidents were seen in women’s football, the rate of completing the game without being substituted was higher than that of men. Obtaining insight into the medical intervention requirements of players during the course of a game will aid in the identification of injury-related behavioral patterns among players.

## INTRODUCTION

Football is the world’s most popular team sport, played by over 250 million people in more than 200 countries. FIFA reported that more than half of the global population aged four and over (more than 3 billion people worldwide), watched the 2018 World Cup on TV. On the other hand, women’s football is also becoming very popular and the number of participants is estimated to reach 40 million world-wide [1, 2].

This popularity has been reflected in the growing importance of international women’s competitions since the first Women’s World Cup held in 1991. It has been estimated that 1.12 billion viewers tuned into official broadcast coverage of FIFA Women’s World Cup 2019 on TV at home, on digital platforms or out-of-home. The 2019 final game was the most watched FIFA Women’s World Cup match ever, with over 260 million viewers and an average live match TV attendance of 82 million, more than double that of the 2015 Women’s World Cup.

Football injuries are common, and the incidence of time-loss injuries in football matches differs for both sexes (Men 31.1 ± 10.8 injuries/1000 h, women 27.6 ± 11.3 injuries/1000 h) [3, 4]. It is known that most of the injuries in professional football occur during competitive games (36 injuries/1000 h) rather than trainings (3.7 injuries/1000 h) [5, 6]. Major sporting events such as the World Cup comprise numerous elite athletes from around the globe, and it is imperative to consider the potential opportunities they present for professionals. From a medical point of view, injuries and incidents that occur in world cup matches can be considered as the most attractive events for sports medicine physicians interested in football medicine. Numerous studies have been conducted on football match injuries in national leagues and international tournaments. These studies generally examine the epidemiology of injuries and the duration of absence (i.e. burden) resulting from these injuries [7–9].

However, the knowledge about field incidents leading to injury time-outs is limited, as well as information on types and characteristics of the related injuries during the match. The incidence of stoppage time due to field injuries during professional football matches has been reported between 1.6 and 3.3 incidents per game for men’s professional football [10–12]. It is important to understand the possible increased effect of football on injuries in elite female players as interest in women’s football develops. However, there is a lack of knowledge about stoppage time injury incidents in women’s matches. A precise understanding of the injury characteristics and circumstances during a game would allow to develop effective prevention and treatment strategies in football medicine as well. Therefore, the aim of the present study was to reveal how often elite level football players need medical care in high-level international tournaments and whether there is any difference between men’s and women’s football.

## MATERIALS AND METHODS

In most studies about football injuries, the medical records of tournament or club medics are used [8, 13–15]. Although these records are not readily accessible, match video records are. Furthermore, the medical records are not able to provide information about injury time-outs during the match. For these reasons, video-based analysis is a frequently preferred method in the current literature [16, 17].

Data were collected retrospectively through video analysis of the 2018 FIFA Men’s World Cup (MWC) matches in Russia and the 2019 FIFA Women’s World Cup (WWC) matches in France. Data on the incident rates during World Cup games were documented through videotape analysis via Wyscout.com^®^. As all data originated solely from publicly available sources and we only report anonymous data, Research Ethics Board approval was not required. Recordings of the television broadcasts for all 116 matches at the MWC 2018 and WWC 2019 were obtained and used for the analysis. Demographic data regarding the World Cup matches is presented in Table 1.

**TABLE 1:** Demographic data for the 2018 FIFA Men’s World Cup (MWC) matches in Russia and the 2019 FIFA Women’s World Cup (WWC) matches in France.

	Men	Women	Total
**Number of matches,** n (%)	64 (55.2)	52 (44.8)	116 (100)

Group matches, n (%)	48 (75.0)	36 (69.2)	84 (72.4)
Qualifying matches, n (%)	16 (25.0)	16 (30.8)	32 (27.6)

**Continent of the teams**, n (%)			

Europe	66 (51.6)	48 (46.2)	114 (49.1)
Africa	15 (11.7)	11 (10.6)	26 (11.2)
Asia	13 (10.2)	14 (13.5)	27 (11.6)
South America	21 (16.4)	10 (9.6)	31 (13.4)
North America	4 (3.1)	14 (13.5)	18 (7.8)
Oceania	3 (2.3)	7 (6.7)	10 (4.3)
Middle America	6 (4.7)	–	6 (2.6)

**Number of matches where the game was stopped,** n (%)	57 (55.3)	46 (44.7)	103 (100)

Two experienced authors reviewed the recordings of all the matches at normal speed, including stoppage times. For accurate analysis, each injury time-outs was reviewed several times at different slowmotion speeds and freeze-frames in a standardized manner. The two authors discussed uncertain situations with each other. Meanwhile, the authors documented all injury time-outs using a dedicated form and subsequently analysed videotape recordings utilising Football Incident Analysis (FIA). [18]. The FIA method is known a reliable tool and has good intra- and inter-observer reproducibility [18]. The injury report contains details of the type, location and mechanism of the injury, game time when the injury occurred, as well as stoppage duration for the incident. It also contains detailed information about the player (age, position, nationality), player’s action during the incident, the score and the teams’ drawing/losing/winning situation at the time of the injury, the game level (group vs. knockout stage), and the medical staff’s decision (substitution or continuation of the play).

An injury time-out was defined as any health complaint that occurred during a match and resulted in the cessation of the game. This included any medical attention received from the medical staff physician and/or physiotherapist, whether on or off the pitch, regardless of the nature/severity of the applied treatment [12]. Injury definitions and recording procedures complied with the International Olympic Committee consensus statement for recording and reporting epidemiological data on injury in sport [19]. Incidence of injuries (injury rate, IR) was expressed as injuries per 1000 player hours of exposure together with the 95% confidence interval (CI).

Sixty-four games were played by 32 teams in the 2018 FIFA Men’s World Cup, whereas 24 teams played a total of 52 matches in the 2019 FIFA Women’s World Cup. The ages of the players and referees obtained and recorded from official data of the Federation Internationale de Football Association (FIFA). The decision made by the referee for each incident was recorded from the video analysis as no foul or a free kick for or against the exposed player and whether the situation resulted in a yellow or red card was also noted. Previous studies have shown that the frequency and type of injury varies according to the minutes of the competition. Therefore, the time of injury was divided into the matches’ 15-minute segments in line with the literature [11, 20].

### Statistical Analysis

The Statistical Package for the Social Sciences (SPSS) software was used for statistical analysis (IBM SPSS Statistics for Mac, Armonk, New York, USA). The variables were analyzed using both visual methods (such as histograms and probability plots) and analytical techniques (such as the Kolmogorov-Smirnov test) to identify normal or non-normal distributions. Descriptive analyses are presented in the form of mean ± standard deviation (SD) and median, minimum, and maximum values for continuous variables and frequency counts and percentages for categorical variables.

Due to the non-normal distribution of the continuous variables, the Mann-Whitney test was used to compare two independent groups. The Chi-squared or Fisher’s Exact Test was used to compare the categoric variables between two independent groups. Effect sizes were measured by Phi coefficient for 2 × 2 Chi-square tests, by Cramer’s V for 2 × 3 Chi-square tests, and by r for the Mann-Whitney U test. Effect sizes were interpreted as follows: < 0.2 null effects, < 0.5 small effects, < 0.8 medium effects, and > 0.8 large effects.

The incidence of injury and injury time-out was calculated using the formula: (number of injuries or injury time-outs × 1000 match hours) / ((minutes of exposure / 60) × number of players exposed), and was given as the number of injuries or injury time-outs per 1000 match-hours with 95% Confidence Intervals (CIs). The injury incidence and injury time-out rates per 1000 match-hours with 95% CI were calculated assuming a Poisson distribution. In order to determine the effect size, the incidence rate ratios (IRRs) with 95% CI and the significant probability of the result of null-hypothesis (incidence rate ratio equals 1) testing for injury incidence rates across the different groups were calculated using Poisson regression models or negative binomial regression models when appropriate. A 5% type-I error level was used to infer statistical significance.

## RESULTS

### Injury time-outs in 2018 FIFA Men’s World Cup and 2019 FIFA Women’s World Cup matches

A total of 265 stoppage times due to field incidents were recorded during both world cups, 123 in the MWC and 142 in the WWC (Table 2). A total of 282 players were treated on the field throughout the study period, including 17 injury time-outs requiring the medical care of two players simultaneously. In 11.2% of the matches (13 out of 116), the referee did not interrupt the game due to the injury incident. The women’s semi-final match recorded the highest number of injury time-outs in a single game (n=8). WWC had an average of 2.7 ± 1.8 injury time-out/match and MWC had an average of 1.9 ± 1.3 injury time-out/match. The incidence of stoppage time per 1000 player hours was significantly higher in women compared to men (81.2 and 56.8 / 1000 match hours, respectively, p = 0.004).

**TABLE 2:** Data on injury time-outs in 2018 FIFA Men’s World Cup (MWC) and 2019 FIFA Women’s World Cup (WWC) matches

	Men	Women	Total	ES	P
**Number of injury time-out**, n (%)		142 (53.6)	265 (100)	–	–

**Total exposure time,** hour	2164.4	1748.3	3912.7	–	–

**Incidence of injury time-out**
Number of injury time-out/1000 player hours (95% CI)	56.8 (47.2 to 66.7)	81.2 (66.1 to 94.8)	67.7 (59.0 to 76.0)	1.42 ^b^ (1.12 to 1.82)	**0.004** ^b^

**Injury time-out duration**, sec
Mean ± SD	101.7 ± 33.0	108.6 ± 42.6	105.4 ± 38.5	0.11	0.1^a^
Median (min-max)	95.0 (45.0–305.0)	101.5 (40.0–332.0)	99.0 (40.0–332.0)		

**Elapsed time between the incident and referee’s whistle**, sec					
Mean ± SD	9.7 ± 15.6	9.1 ± 15.5	9.4 ± 15.5	0.08	0.3^a^
Median (min-max)	5.0 (1.0–128.0)	4.0 (1.0–124.0)	4.0 (1.0–128.0)		

**Timing of injury time-out in the match**, n (%)					
0–15 min	10 (8.1)	11 (7.7)	21 (7.9)	0.09	0.9^c^
15–30 min	18 (14.6)	23 (16.2)	41 (15.5)		
30–45 min	25 (20.3)	31 (21.8)	56 (21.1)		
45–60 min	20 (16.3)	22 (15.5)	42 (15.8)		
60–75 min	20 (16.3)	23 (16.2)	43 (16.2)		
75–90 min	27 (22.0)	25 (17.6)	52 (19.6)		
Stoppage time	3 (2.4)	7 (4.9)	10 (3.8)		

**Time of injury time-out in the match (halves of the game)**, n (%)					
First half	53 (43.1)	65 (45.8)	118 (44.5)	0.08	0.5 ^c^
Second half	67 (54.5)	70 (49.3)	137 (51.7)		
Extra time	3 (2.4)	7 (4.9)	10 (3.8)		

**Number of players that were treated on the field**, n (%)	132 (46.8)	150 (53.2)	282 (100)	–	–

**Field zone**, n (%)					
1	57 (43.2)	60 (40.0)	117 (41.5)	0.12	0.3^c^
2	45 (34.1)	56 (37.3)	101 (35.8)		
3	30 (22.7)	30 (20.0)	60 (21.3)		
Uncertain	–	4 (2.7)	4 (1.4)

**Action of the injured player**, n (%)					
Defending	78 (59.1)	95 (63.3)	173 (61.3)	0.08	0.5^c^
Attacking	51 (38.6)	54 (36.0)	105 (37.2)		
Uncertain	3 (2.3)	1 (0.7)	4 (1.4)		

**Score during injury time-out**, n (%)					
Drawing	63 (47.7)	69 (46.0)	132 (46.8)	0.05	0.7^c^
Winning	41 (31.1)	43 (28.7)	84 (29.8)		
Losing	28 (21.2)	38 (25.3)	66 (23.4)		

**Referees' decision**, n (%)					
Continue	98 (74.2)	108 (72.0)	206 (73.0)	0.05	0.7^c^
Foul	31 (23.5)	40 (26.7)	71 (25.2)		
Advantage	3 (2.3)	2 (1.3)	5 (1.8)		

**Referees' card decision**, n (%)					
None	120 (90.9)	128 (85.3)	248 (87.9)	0.09	0.2^d^
Yellow	12 (9.1)	22 (14.7)	34 (12.1)		
Red	–	–	–		

**Injured player's participation in the game**, n (%)					
Substituted	36 (27.3)	23 (15.3)	59 (20.9)	0.15	0.02^d^

a : Mann-Whitney U Test,

b : Poisson regression model IRR (95% CI),

c : Pearson Chi-Square Test,

d : Fisher’s Exact Test, ES: Effect size.

The duration of a stoppage time due to an injury was determined through videotape analysis and is defined as the interval between two whistles of the referee. This is because the referee stops the game in order to invite medical staff onto the pitch and the game starts again after the potential needed medical check and treatment. The mean stoppage time was 105.4 ± 38.5 seconds (40–332 sec.) which was not different between men’s and women’s football (Table 2). The average time from the incident to the referee’s whistle to stop the game for the injury event was 9.4 ± 15.5 seconds (minmax: 1.0-128.0 sec.).

The frequency of time-outs was slightly higher during the second half of the games. (Table 2). Upon further analysis of the data based on six quarter-hour intervals, we observed that the incidence of injury time-outs was higher during the final 15 minutes of both halves. Half of the total game duration was tied (draw), equating to 50.3% (5,375 minutes) of the entire matches duration. Players received medical care 132 times, representing approximately half of the total events (46.8%), during periods when the score of the game was tied. The team leading (n=84, 29.8%) at the time of the incident had more injury time-outs than the losing team (n=66, 23.4%). A review of the incidents that occurred in the final 15 minutes of the match (n=52) revealed that 50% were taken by the leading team (n=26), 26.9% by the losing team (n=14) and 23.1% by tied-score team (n=12).

Almost one sixth of all incidents caused the injured women players to be substituted (n=23, 15.3%) (Table 2). Despite women needed more medical care during games, the substitution rate was found higher in men (n = 36, 27.3%, p = 0.02).

### Mechanism, body regions, types and incidence of injuries that caused injury time-out

**FIG. 1:**
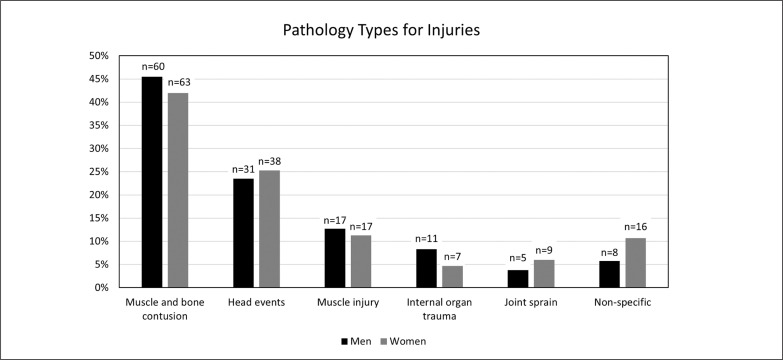
Pathology-types for injuries leading to injury time-outs.

Injured women were younger than injured men (27.5 ± 3.4 years (min-max: 19.0–39.0 years), 28.6 ± 4.1 years (min-max: 20–45 years), p = 0.007, respectively). The most common incident mechanism leading to the need for medical care for both women and men was sudden-onset contact-, compared to sudden-onset non-contact injuries (n = 119, 79.3%, n = 100, 75.8%, respectively) (Table 3). The most common injured site was the lower extremity in men (47.7%) and women (46.0%), followed by the head and neck, trunk and upper extremity, respectively. Ankle and lower leg were the most commonly injured body parts in the lower extremity in both sexes. The types of injuries are presented in Figure 1.

**TABLE 3:** Mechanism of injuries and body region and areas for injuries.

	Men (n = 132)	Women (n = 150)	Total (N = 282)
**Mechanism of injury, n (%)**			
Sudden-onset non-contact	19 (14.4)	19 (12.7)	38 (13.5)
Sudden-onset contact	100 (75.8)	119 (79.3)	219 (77.7)
Recurrent	4 (3.0)	–	4 (1.4)
Uncertain	9 (6.8)	12 (8.0)	21 (7.4)

**Body regions and areas for injuries, n (%**)			
Head and neck	32 (24.2)	39 (26.0)	71 (25.2)
Head	31 (23.5)	38 (25.3)	69 (24.5)
Neck	1 (0.8)	1 (0.7)	2 (0.7)
Upper limb	8 (6.1)	8 (5.3)	16 (5.7)
Shoulder	4 (3.0)	5 (3.3)	9 (3.2)
Elbow	2 (1.5)	–	2 (0.7)
Wrist	2 (1.5)	3 (2.0)	5 (1.8)
Trunk	22 (16.7)	19 (12.7)	41 (14.5)
Chest	8 (6.1)	6 (4.0)	14 (5.0)
Thoracic spine	1 (0.8)	2 (1.3)	3 (1.1)
Lumbosacral	2 (1.5)	4 (2.7)	6 (2.1)
Abdomen	11 (8.3)	7 (4.7)	18 (6.4)
Lower limb	63 (47.7)	69 (46.0)	132 (46.8)
Hip/groin	11 (8.3)	2 (1.3)	13 (4.6)
Thigh	8 (6.1)	9 (6.0)	17 (6.0)
Knee	11 (8.3)	13 (8.7)	24 (8.5)
Lower leg	15 (11.4)	18 (12.0)	33 (11.7)
Ankle	18 (13.6)	27 (18.0)	45 (16.0)
Unspecified	7 (5.3)	15 (10.0)	22 (7.8)

### Incidence of injuries that caused injury time-out

Women had a higher total injury incidence compared to men (IRR = 1.4 [95%CI = 1.1–1.8], p = 0.005) (Table 4). When the incidence of injuries by body region was compared, it was observed that this difference was due to the unspecified body region as opposed to the specific body region (IRR = 2.7 [95%CI = 1.1–6.5], p = 0.03). A total of 34 players (12.1%) received medical care for muscle injuries in both tournaments (men: n = 17, 12.9%; women: n = 17, 11.3%) (15). The incidence of structural muscle injuries was slightly higher in women (9.7 / 1000 matches/hour and 7.9 / 1000 matches/hour, respectively), however, there was no significant difference between the two groups (IRR = 1.2 [95%CI = 0.6–2.4], p = 0.5).

**TABLE 4:** Injury incidences according to body region and areas.

	Men		Women		Total		IRR (95% CI)	P value
**Total exposure time, hour**	2164.4		1748.3		3912.7			

	**n**	**Incidence (95% CI)**	**n**	**Incidence (95% CI)**	**n**	**Incidence (95% CI)**		

**Total injury**	132	61.0 (50.8 to 71.7)	150	85.8 (69.3 to 100.7)	282	72.1 (62.7 to 81.1)	1.4 (1.1 to 1.8)	**0.005**

**Head and neck**	32	14.8 (9.6 to 20.7)	39	22.3 (15.0 to 29.3)	71	18.2 (13.9 to 22.7)	1.5 (0.9 to 2.4)	0.09

**Upper limb**	8	3.7 (0.9 to 6.5)	8	4.6 (1.5 to 7.5)	16	4.1 (2.0 to 6.1)	1.2 (0.5 to 3.3)	0.7

**Trunk**	22	10.2 (5.7 to 14.6)	19	10.9 (5.6 to 16.0)	41	10.5 (7.1 to 13.8)	1.1 (0.6 to 2.0)	0.9

**Lower limb**	63	29.1 (21.8 to 36.0)	69	39.5 (29.1 to 48.7)	132	33.7 (27.5 to 39.3)	1.4 (1.0 to 1.9)	0.09

**Unspecified**	7	3.2 (0.6 to 6.1)	15	8.6 (3.3 to 13.9)	22	5.6 (2.9 to 8.5)	2.7 (1.1 to 6.5)	**0.03**

Total exposure time was calculated using the formula: (number of players exposed on the field) × (number of game-hours that the team has played). The incidence of injury was calculated using the formula: ((number of injuries × 1000 match hours) / total exposure time). Injury incidence was given as the number of injuries per 1000 match-hours with 95% Confidence Intervals (CIs). All of the analyses were performed using Poisson regression. Incidence rate ratios (IRRs) were calculated as the incidence of female to incidence of male (Reference group: male).

### Total injury incidences for continents

According to the analysis of total injury incidences by teams’ continents, the incidence of receiving medical care during play was found to be significantly higher in African players compared to other continents for both genders (Incidence [95%CI]: Africa = 126.0 [92.8 to 159.3], Europe = 68.7 [56.7 to 83.7], South America = 51.8 [27.4 to 75.3], North America = 30.3 [2.5 to 58.1], p < 0.001) (Table 5).

**TABLE 5:** Total injury incidences by teams’ continents.

N = 282	Exposure time, hour	n	Incidence (95% CI)	IRR (%95 CI)	P Value
**Africa**	428.6	54	126.0 (92.8 to 159.3)	1.00 (reference)	
**Europe**	1951.2	134	68.7 (56.7 to 83.7)	0.55 (0.40 to 0.75)	**< 0.001**
**Asia**	445.5	38	85.3 (52.6 to 118.1)	0.68 (0.45 to 1.03)	0.06
**South America**	521.1	27	51.8 (27.4 to 75.3)	0.41 (0.26 to 0.65)	**< 0.001**
**North America**	297.0	9	30.3 (2.5 to 58.1)	0.24 (0.12 to 0.49)	**< 0.001**
**Oceania**	170.2	11	64.6 (34.0 to 93.5)	0.51 (0.27 to 0.98)	**0.04**
**Middle America**	99.0	9	90.9 (24.2 to 157.6)	0.72 (0.36 to 1.46)	0.4

The exposure time was calculated using the formula: (number of players exposed on the field) × (number of game-hours that the team has played). The incidence of injury was calculated using the formula: ((number of injuries × 1000 match hours) / total exposure time). Injury incidence was given as the number of injuries per 1000 match-hours with 95% Confidence Intervals (CIs). All analyses were performed using Poisson regression. Incidence rate ratios (IRR) were calculated with reference to the incidences of African teams.

## DISCUSSION

We investigated the stoppage times due to injury incidents resulting in players receiving primary medical care on the field in professional football matches in elite level tournaments. This is the first study comparing men and women.

We showed that women had a higher incidence of injury time-outs compared to men. One of the main findings of our study is that women had a higher incidence of injury time-out compared to men. Previous studies have found that the overall injury incidence was significantly higher in men than in women, however the rate of serious injuries (causing more than 28 days of absence) appears to be significantly higher in women [4, 21–24]. It would be expected to see higher incidents in men’s football, since the men’s game may have greater frequency and force of physical contact as it involves larger, faster players on the same-sized field. However, our findings clearly show that women’s professional football caused more incidents than men’s in recent world cup tournaments. This may be due to gender differences in the experience of pain, which are multifactorial and depend on complex factors such as psychosocial factors and gonadal hormone levels [25]. In this context, the results of previous studies showed that women stay away from the pitch for longer periods due to on-field injuries [4, 21–24]. Another finding of our study that supports this might be that although women needed more frequent medical care during games, the rate of substitution rate due to field injuries was lower than in men. We recognize that the factors underlying sex differences in pain experience are multifactorial and complex; therefore, further investigation of the reasons for the higher incidence of injuries in women’s football and a better understanding of risk factors (such as psychosocial factors, gonadal hormone levels, and menstrual phase) are needed in order to develop effective strategies for injury prevention.

Although football injuries are of equal concern for men and women, most of the data reported to date relate to men. To the best of our knowledge, only one study has been published comparing data between men and women in top-level international tournaments [26]. In contrast to our study, Walden et al. compared the difference of time-loss injuries which were defined as an incident causing a player to miss the next training session or match by analyzing the exposure and injury characteristics of European Football Championships between men and women players, and youth players as well. Their findings revealed that the injury incidences at the 2004 Men’s European Championships and the 2005 Women’s European Championship were similar between sexes concluding that the risk of injury in international football is not higher in women than in men [26]. In our study, the total match injury incidence was significantly higher in women’s football. Herein, we agree with the conclusion of Giza et al. which emphasized that age, skill level or improved training and fitness may have influenced injury incidence, adding a clear sex dimension to this multifactorial approach proposal [23]. The results of the previous study showed that the injury time-outs seem to be higher in the Turkish First Division compared to the Turkish Super League (3.98/game vs 3.14/game respectively) [12]. In accordance with the previous literature, the skill level has definitely an effect on match injury incidence, but the role of sex should not be ignored and should be taken into account [12, 26, 27].

Previously, the average effective playing time ranged between 52 and 58 minutes per match, during the 2018 FIFA World Cup in Russia males FIFA world cup. This indicates that a significant portion of the playing time is lost in various ways. We showed that field injuries resulted in an average of 3 minute and 15 second stoppage time in the 2018 FIFA World Cup. However, the total stoppage time was found higher in 2019 Women’s World Cup (on average, 4 minutes 56 seconds). Given the competitive nature of football, particularly at the highest levels, it is plausible that changes in the score could influence a number of factors related to the conduct of the game. These include the strategies employed by the teams, the attitudes and precautions of the players, the intensity of the match, and/or the incidence of injury. The main conclusion drawn from this study that supports this idea is the association between injury breaks in World Cup matches and score adjustments. A comparison of the medical care required by players revealed that those of the currently winning team required significantly more medical care than those on the losing team, aligning with the study of Ryynänen et al. [28]. However, as expected, teams leading at the incident time had a significantly higher incidence of injury time-outs than teams that were losing during the last 15 minutes of the game (50.0% vs. 26.9%, respectively). We contend that feigning injury may be a more common tactic for cooling down a match than previously assumed. In general, when their team is leading, players are tempted to burning time off the clock while taking a brief respite. Herein, we may conclude that, almost 5 minutes of stoppage time due to injuries may affect the attractiveness of a football match, especially considering that the most of these incidents seem questionable or unnecessary.

Another interesting finding of our study was about the lower substitution rates in women football due to these incidents. The literature indicates that the overall injury incidence was reported to be similar in men and women football, although the proportion of severe injuries has been shown to be higher in women [21, 29]. However, we found that the rate of substitutions after the incidents in men was significantly higher than women (27.3% vs 15.3%, respectively). This result aligns with the results of our previous study in men footballers, with 17.4% of incidents resulting in an impossibility to complete the game in Turkish Super League [12]. Given the short-term nature of the tournament in question, it was impossible to ascertain whether the players who could not complete the game could participate in the potential subsequent ones. Therefore, the information about the time for return to play was not obtained. Although the latter information would be important to capture, our study setting (observational retrospective study based on publicly available data) does not allow it. However, higher rates of substitutions after men’s injuries may be possibly attributed to (i) potentially higher damage/more serious injuries occurring during the events, and/or (ii) less questionable incident frequency in men’s world cup tournament. The latter relates to some cases where the player would simulate an injury for a particular reason [12]. Alinged with the above discussed point (i) the higher rates of sudden onset non-contact injuries in men’s football (14.4% vs 12.7%, respectively) might be one of the contributing factors for increased substitutions frequency compared with women. The risk of sustaining an injury without contact with another player (eg. muscle injuries) is thus high in international football and may also be of importance for suggesting an increase in substitution rate by the governing bodies (e.g., FIFA). In that regard, FIFA has recently considered an additional substitution when a player is taken out of the field by the medical staff for a suspected concussion. In that regard, we suggest to enlarge this measure and consider allowing more ‘injury related substitution’ for the fairness of the game, even though we reconsider that this point is tricky, as simulating an injury by unfair players/teams is something not easy to objectively assess from a referee’ prospective. As we know, most injuries in football were sustained during contact with another player regardless of the sex [4, 30]. However, why match incidents caused higher substitution rates for men’s foot-ball remains to be answered.

Regarding the location of the injuries, the literature reported that the most common incident location in football is the lower limb, with lower limb injuries accounting for 74% to 86.8% of total injuries [21, 31, 32]. Accordingly, we found that the rate of lower limb events was predominant (approximately half of all events in both sexes. Women have a different injury risk profile then men and studies have reported that the risk of serious knee injury (such as anterior cruciate ligament (ACL) rupture) is at least twice as high in women, regardless of level of exposure or participation [21, 32, 33]. However, as mentioned above, since we evaluated a short-term tournament and it was not possible to determine the exact diagnoses of the players’ injuries by video analysis, our study could not provide any information about the incidence of specific diagnoses such as ACL injury.

The unpublished results of our group’ previous study revealed that the player’s nationality is another reason for injury occurrence [12]. European citizen football players were found to receive significantly less medical care than Turkish counterpart when compared to their time on the pitch, while for African and South American players these rates were consistent with the time spent on the pitch [12]. In the present study, it is noteworthy that the incidence of receiving medical care during World Cup matches was significantly higher in African players (Middle America being ranked second here) for both genders. Rosenbaum et al. stated that the assumptions regarding the likelihood of players from certain cultures or regions faking injuries should be made with caution for such tournaments since the number of countries representing each confederation is small compared to the sample size of games evaluated [34]. This points fully applies here with both above mentioned regions (Africa and Middle America), being only a small portion of the tournaments’ teams. We believe that these data are important for football governing bodies not only in deciding whether efforts to prevent injury simulation are necessary, but also in deve loping prevention strategies.

There are limitations to our study. First, we recorded injuries as events where a player required medical treatment from a team physician and/or physiotherapist, but the only source of information we obtained was videotape recordings. Therefore, we were unable to define what the definitive diagnosis of injuries was or what the exact cause of a substitution was. However, we identified all match incidents on the video recordings in top level tournament and therefore the completeness of data and the level of competition chosen can be considered strong points of the present study. Secondly, as we evaluated a short-term tournament; it was not possible to identify whether all of these players who could not complete the game were able to play next game, or train the day after. Third, comparing our findings with previous literature should be done with caution, as risk factors and inter-player competition may differ between domestic league games and elite level international tournaments.

## CONCLUSIONS

This is the first study that compared the incidence and features of stoppage time due to field injuries between men’s and women’s professional football in highest-level tournaments (FIFA Men’s World Cup and FIFA Women’s World Cup). Both injury time-out incidence and overall injury incidence were higher in women’s games than in men’s. The expansion of the knowledge of team physicians, coaches, referees, and the football governing bodies regarding the medical requirements of players during a game would potentially facilitate the identification of player behavior patterns and the promotion of fair play. Not only we provide information for football medicine physicians about field injuries, but we also hope that our study will potentially impact awareness regarding referees’ attitudes towards foul play.
